# Composites of Poly(3-hydroxybutyrate) and Mesoporous SBA-15 Silica: Crystalline Characteristics, Confinement and Final Properties

**DOI:** 10.3390/polym16081037

**Published:** 2024-04-10

**Authors:** Tamara M. Díez-Rodríguez, Enrique Blázquez-Blázquez, Ernesto Pérez, María L. Cerrada

**Affiliations:** Instituto de Ciencia y Tecnología de Polímeros (ICTP-CSIC), Juan de la Cierva 3, 28006 Madrid, Spain; t.diez@ictp.csic.es (T.M.D.-R.); enrique.blazquez@ictp.csic.es (E.B.-B.); ernestop@ictp.csic.es (E.P.)

**Keywords:** poly(3-hydroxybutyrate) (PHB), mesoporous SBA-15 silica, composites, confinement, thermal properties, synchrotron SAXS, DMTA

## Abstract

Several composites based on poly(3-hydroxybutyrate) (PHB) and mesoporous SBA-15 silica were prepared by solvent-casting followed by a further stage of compression molding. The thermal stability, phase transitions and crystalline details of these composites were studied, paying special attention to the confinement of the PHB polymeric chains into the mesopores of the silica. For that, differential scanning calorimetry (DSC) and real-time variable-temperature X-ray scattering at small angles (SAXS) were performed. Confinement was stated first by the existence of a small endotherm at temperatures around 20 °C below the main melting or crystallization peak, being later confirmed by a notable discontinuity in the intensity of the main (100) diffraction from the mesoporous silica observed through SAXS experiments, which is related to the change in the scattering contrast before and after the crystallization or melting of the polymer chains. Furthermore, the usual α modification of PHB was developed in all samples. Finally, a preliminary investigation of mechanical and relaxation parameters was carried out through dynamic–mechanical thermal analysis (DMTA). The results show, in the temperature interval analyzed, two relaxations, named α and β (the latest related to the glass transition) in order of decreasing temperatures, in all specimens. The role of silica as a filler is mainly observed at temperatures higher than the glass transition. In such cases, stiffness is dependent on SBA-15 content.

## 1. Introduction

There is, nowadays, a big concern about sustainability and preservation of the environment, both by demands of society and by the increasingly restrictive directives on environmental issues. For these reasons, the use of commodity plastics, widespread for decades, is being reduced especially for single-use applications: packaging, catering or agriculture, among others. Moreover, an efficient recycling of these actual polymer wastes is mandatory.

A suitable alternative for commodities is the use of biodegradable polymers, but unfortunately, most of them have significant disadvantages, precluding their massive usage in areas demanding great consumption.

Polyhydroxyalkanoates (PHA), which are biopolyesters of bacterial origin, are a very remarkable biodegradable and biobased polymer family. Among them, poly(3-hydroxybutyrate) (PHB) and their copolymers with 3-hydroxyvalerate (PHBHV) are the best-known systems. In fact, PHB displays certain performances that are similar to those in some commodities, showing, however, some practical deficiencies [1,2,3,4] that prevent its full attractiveness. Thus, high manufacture prices stand out together with a low toughness arising from its high crystallinity, embrittlement effects with time owing to secondary crystallization, and also a narrow processing window because of its prompt thermal degradation at relatively low temperatures [5].

In order to overcome these drawbacks, several strategies have been proposed. These approaches include the addition of nucleating or plasticizing agents (or both), a mixture with other polymers, and the use of adequate fillers [6,7]. Concerning the latter, mesoporous silicas are especially versatile among the wide variety of materials used as fillers, with the added advantage of involving nanodimensions. These silicas were first disclosed by Exxon Mobil in 1992 [8]. The Mobil Crystalline Materials No. 41, labeled commonly as MCM-41, is characterized by showing a hexagonal arrangement with parallel one-dimensional pores of a diameter of circa 3 nm. Years later, alternative mesoporous silicas were synthesized in 1998 at the Santa Barbara University of California. Among them, the best-known member is the Santa Barbara Amorphous SBA-15, displaying also a hexagonal array similar to that exhibited by MCM-41, but now the pore diameters range from around 5 to 10 nm [9]. These silicas are, therefore, nanostructurally ordered. Moreover, they display rather interesting properties, including their easy functionalization, so that they are being used in different fields, like catalysis, coatings, cosmetics, diagnostics, drug delivery, gas and bio-separation, optics and nanotechnology, among others.

Another very remarkable aspect of these materials is the fact that polymeric chains might go inside the nanometric channels existing in the silica mesostructure, thus leading to their confinement. This confinement has been observed in several systems [10,11,12,13], initially detected by Differential Scanning Calorimetry (DSC). Later on, experiments of Small Angle X-ray Scattering with synchrotron radiation (SAXS) were described as a very useful and conclusive tool. Thus, confinement, which is dependent on polymer molecular weight [14] and on mesoporous pore size [15], is characterized in real-time variable-temperature SAXS measurements through a notable discontinuity in the intensity of the primary (100) diffraction of the mesoporous silica, which is related to the change in the scattering contrast before and after the melting of the polymer chains [16,17].

The evaluation of confinement of organic solids in glasses with controlled pores has been an interesting issue for a long time [18,19]. The development of those crystals with restricted dimensions can be initially described by Gibbs theories [20], which were further adapted to small crystals in confined geometries, leading to the well-known Gibbs–Thomson equation [21,22].

The advent of nanotechnology has led to a renewed interest in the study of confinement effects, with the final purpose of analyzing the influence of such confined crystals on the final bulk properties, which is especially interesting in polymeric systems [12]. In addition, the macromolecular chains that may be partially inserted into the pores of the mesoporous structures can be of capital importance for obtaining polymeric nanocomposites, which in turn can influence the final performance of those polymeric systems.

The aim of the present study is the preparation and characterization of composites based on PHB and different contents of SBA-15 silica, in order to analyze their crystalline characteristics, the existence (or absence) of confinement of PHB chains into the pores of the silica, and, preliminarily, their stiffness as well as relaxations characteristics. For that, differential scanning calorimetry (DSC) and real-time variable-temperature X-ray scattering at both small angles (SAXS) and wide angles (WAXS) were performed, with the SAXS experiments especially suited for verifying the existence of confinement in the composite materials. Finally, an initial estimation of values for storage modulus, as a mechanical parameter, and of the different relaxation processes was carried out through dynamic–mechanical thermal analysis (DMTA).

## 2. Materials and Methods

### 2.1. Materials and Chemicals

A commercially available PHB (purchased from Ercros, Barcelona, Spain) with an MFI of about 30 g/10 min at 190 °C with a load of 2.16 kg, was used as polymeric matrix in this research. SBA-15 particles were purchased from Sigma-Aldrich, St. Louis, MO, USA (specific surface area, S_BET_ = 517 m^2^/g; total pore volume, V_t_ = 0.83 cm^3^/g; average mesopore diameter, D_p_ = 6.25 nm) [23] and were used as received.

### 2.2. (Nano)composite and Film Preparation

Composites of PHB with different contents of SBA-15 particles (nominal amounts of 2, 4, 8 and 16% by weight) were prepared. They were labeled as PHBSBA2, PHBSBA4, PHBSBA8 and PHBSBA16, respectively. Drying of the components was carried out prior to their obtainment. The PHB was placed in an oven at 50 °C for 20 min followed by a drying under vacuum also at 50 °C for 20 h. The SBA-15 particles were dried at 100 °C for 24 h under vacuum. After this drying stage, the protocol followed was: a suitable content of SBA-15 silica was dispersed in chloroform at the same time that a PHB/chloroform solution (6 wt.% in PHB) was prepared. Both dispersion and solution were stirred for 18 h at room temperature. Afterward, the dispersion containing the silica particles was added to the PHB/chloroform solution. This PHB/silica/chloroform dispersion was stirred for a further 6 h at room temperature before it was poured into Petri dishes and dried at room temperature for 48 h. The resultant composite films were additionally dried in a vacuum oven at 85 °C for 2 h. A sample of neat PHB was also prepared with that same protocol.

These materials were subsequently processed by compression molding in a hot plate Collin press, New York, NY, USA. Initially, the material was maintained at a temperature of 200 °C, firstly without pressure for 2 min and, later, under a pressure of 30 bar for 4 min. Afterward, a cooling process at a relatively rapid rate of around 80 °C/min and at a pressure of 30 bar was applied to the different composites from their molten state to room temperature.

### 2.3. Transmission Electron Microscopy

Measurements were performed at room temperature in a 200 kV JEM-2100 JEOL microscope (Tokyo, Japan). The particles were dispersed in acetone in an ultrasonic bath for 5 min and then deposited in a holder prior to observation.

### 2.4. Scanning Electron Microscopy

Images of scanning electron microscopy (SEM) were attained in a Philips XL30 microscope (Amsterdam, The Netherlands). The samples were coated with a layer of 80:20 Au/Pd alloy and deposited in a holder before visualization.

### 2.5. Thermogravimetric Analysis

Thermogravimetric analyses (TGA) were carried out using TGA 2 equipment from METTLER TOLEDO (Columbus, OH, USA) at a heating rate of 10 °C/min under a nitrogen atmosphere. From these experiments, both the degradation behavior of the different samples and the particular SBA-15 amount incorporated into the composites during extrusion were estimated.

### 2.6. Differential Scanning Calorimetry

Calorimetric experiments were performed in a TA Instruments Q100 calorimeter connected (New Castle, DE, USA) to a cooling system and calibrated with different standards. The sample weights were around 3 mg. A heating–cooling–heating cycle was used, with a scanning rate of 20 °C/min in the temperature range from −50 to 200 °C. The crystallinity was estimated by considering a melting enthalpy of the 100% crystalline PHB of 146 J/g [24].

### 2.7. X-ray Experiments with Synchrotron Radiation

Simultaneous real-time variable-temperature SAXS/WAXS experiments were carried out with synchrotron radiation in beamline BL11-NCD-SWEET at ALBA (Cerdanyola del Vallès, Barcelona, Spain) at a fixed wavelength of 0.1 nm. A Pilatus detector was used for SAXS (off beam, at a distance of 294 cm from the sample) and a Rayonix one for WAXS (at about 12 cm from the sample, and a tilt angle of around 29 degrees). A Linkam Unit, connected to a cooling system of liquid nitrogen, was employed for the temperature control. The calibration of spacings was obtained by means of silver behenate and Cr_2_O_3_ standards. The initial 2D X-ray images were converted into 1D diffractograms, as a function of the inverse scattering vector, s = 1/d = 2 sin θ/λ, by means of pyFAI python code (ESRF), modified by ALBA beamline staff. Film samples of around 5 mm × 5 mm × 0.2 mm were used in the synchrotron analysis.

### 2.8. Dynamic Mechanical Thermal Analysis (DMTA)

Dynamic Mechanical Thermal Analyses were carried out in a TA Q800 Dynamic Mechanical Thermal Analyzer (New Castle, DE, USA), working in a tensile mode. The storage modulus, E′, loss modulus, E″, and the loss tangent, tan δ, of PHB and its composites with SBA-15 were determined as functions of temperature over the range from −55 to 170 °C, at fixed frequencies of 0.5, 1, 3 and 10 Hz, and at a heating rate of 1.5 C/min. Strips of 2.2 mm wide and 15 mm long, cut from the compressed-molded films, were used for this analysis. Composite PHBSBA16 was not possible to analyze, since it is too rigid and fragile. The viscoelastic relaxations were determined from these studies. A smoothing function using an FFT filter with three points was applied to the loss modulus curves at different frequencies.

## 3. Results and Discussion

### 3.1. Morphological Characteristics

**Figure 1:**
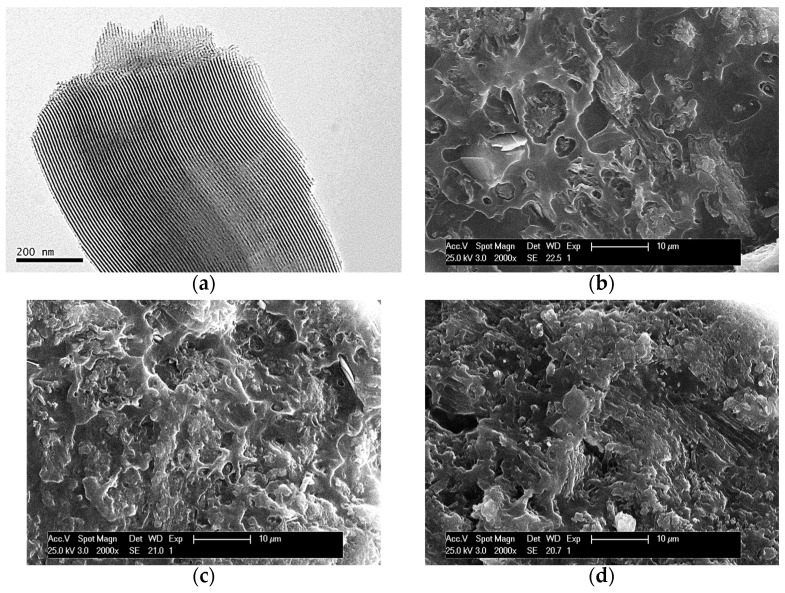
TEM micrographs of SBA-15 particles, dispersed in acetone and further deposited in a holder (**a**). SEM pictures at fracture surface for different composites: PHBSBA4 (**b**), PHBSBA8 (**c**), and PHBSBA16 (**d**).

Figure 1a displays the TEM micrograph of the SBA-15 particles used for preparing the composites. This picture allows observation of the interior of particles, showing the existence of their ordered arrangements in a hexagonal morphology as well as the long parallel channels that constitute that particular ordering.

SEM pictures at fracture surfaces of different composites are depicted in Figure 1b–d. The average size of an individual SBA-15 particle is around 350 nm wide and 0.9 μm long [13]. Accordingly, a suitable dispersion of SBA-15 particles was obtained within the PHB matrix since there is no observation of detectable bulky inorganic domains across the materials at the different mesoporous silica contents. These results seem to indicate that the preparation protocol followed has allowed a rather good contact at interfaces between silica particles and the PHB chains. On the other hand, a change is also noted in the PHB surface, from relatively ductile to rather fragile as the amount of SBA-15 in the composites increases. This fact is deduced from the differences in the fracture surface. This is rather continuous in the PHBSBA4 composite while it becomes coarser in the PHBSBA8 material and even more in the PHBSBA16 one. The appearance of coarseness in the fracture surface is characteristic of fragility. This fragility will be further discussed.

### 3.2. Thermal Stability

**Figure 2:**
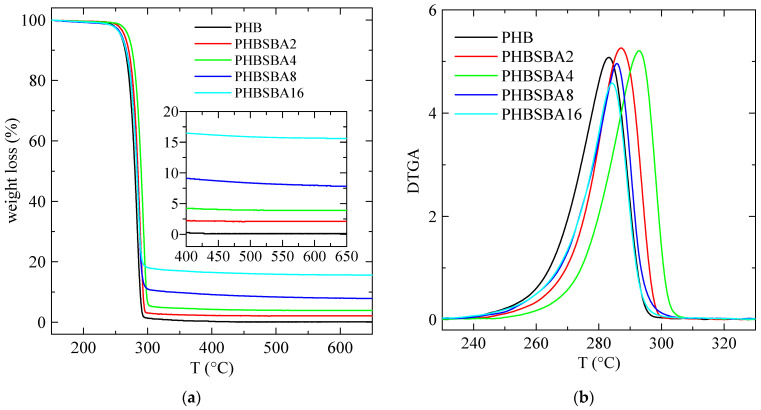
TGA (**a**) and DTGA (**b**) curves under nitrogen for PHB and its composites with SBA-15. The amplified inset in (**a**) reflects the content in SBA-15 of the composites.

The TGA curves, under a nitrogen environment, for the different samples are shown in Figure 2a. These curves indicate that a single primary stage is present in the decomposition of all the specimens, with an inflection point of about 290 °C. Furthermore, they also display a final inert residue, which allows determining the actual SBA-15 content in the samples. This content is collected in Table 1, compared with the nominal values.

Moreover, the DTGA curves, depicted in Figure 2b, are characteristic of that single decomposition stage, and the corresponding values of T_max_ are deduced from them, being also collected in Table 1. These values are close to others reported before in the literature [1].

Although mesoporous silicas were also used, sometimes, as catalysts for thermal decomposition [25,26], however the results in Figure 2 and Table 1 indicate a slightly higher thermal stability in the composites. In fact, the value of T_max_^DTGA^ increases first in composites PHBSBA2 and PHBSBA4, then decreases for the other two composites, although the values of T_max_^DTGA^ are always higher than the ones for neat PHB. This is a behavior found in some polymer composites: a certain property may display better performance than the neat polymer at low filler contents, but when the content increases, at some point the property begins to weaken somewhat, probably due to the formation of some aggregates that are hindered at smaller filler amounts.

### 3.3. DSC Studies: Phase Transitions and Confinement of PHB Chains

**Figure 3:**
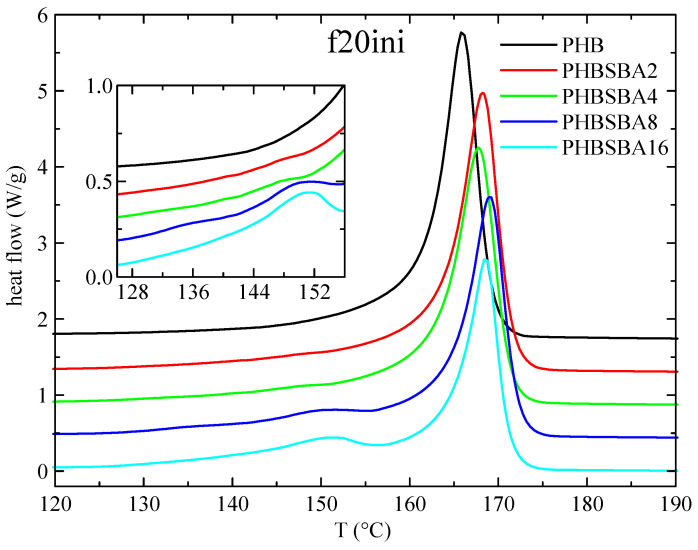
DSC heating curves (endo up) for PHB and its composites with SBA-15 in the first melting at 20 °C/min. The inset shows the amplification in the region of the melting of confined crystals of PHB.

DSC experiments were used, first, to determine the different phase transitions. Thus, Figure 3 shows the DSC heating curves obtained in PHB and its composites with SBA-15 during the first melting at 20 °C/min. Two clear temperature intervals are observed: a main melting endotherm, at around 166 °C for the neat PHB with slightly higher values for the composites, and a second interval at lower temperatures (between around 130 and 155 °C). No features are observed for neat PHB in this inferior interval, but one or two small melting endotherms are observed in the composites, involving an enthalpy that increases with the SBA-15 content, as observed in the amplified inset.

These small endotherms are attributed, eventually, to the melting of the PHB crystallites that are confined inside the pores of SBA-15. Since these crystallites shall be much thinner than the ones outside of the mesoporous silica particles, their melting temperature is going to be then considerably lower than that of the PHB crystals outside the channels. The reason for that is found when considering the Gibbs–Thomson equation, mentioned above. Thus, a simplified equation can be considered [27,28,29] in the case of “regular” lamellar crystals, but a more general form [30,31] is required for the thinner crystallites confined in the mesoporous silica channels, which are supposed to present very low values in their lateral size, restricted by the pore diameter. Consequently, significantly depressed melting temperatures shall be exhibited by those confined crystals.

Moreover, it seems obvious that the more SBA-15 silica is present in the composites, the more channels will be available for confinement, and, therefore, the enthalpy involved in the melting of these confined crystallites will increase. Regarding the fact that two components seem to be observed in the melting of the confined crystallites, we will come back below to this issue.

**Figure 4:**
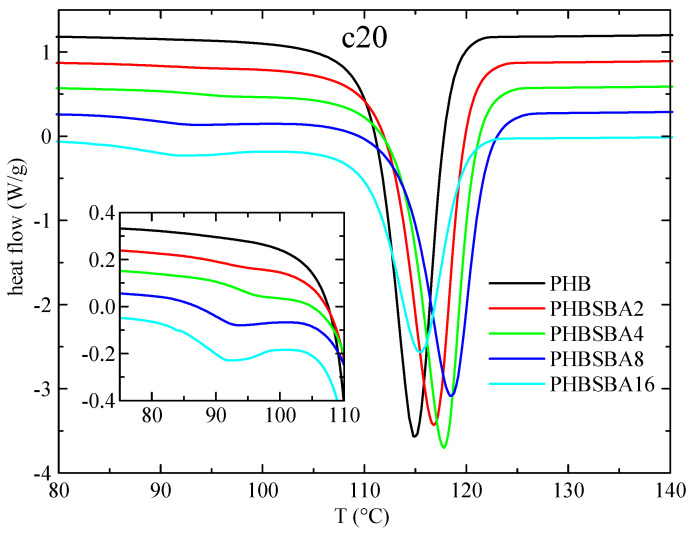
DSC curves (exo down) for PHB and its composites with SBA-15 in the cooling from the melt at 20 °C/min. The inset shows the amplification in the region of the crystallization of confined PHB.

Figure 4 shows the DSC curves for PHB and its composites in the subsequent cooling from the melt (at 20 °C/min). It is important to emphasize at this point that the PHB samples do not crystallize on cooling from the melt at regular DSC rates in most of the published works, most probably because the molecular weight is too high, and only in a few cases [1,32] that crystallization is readily observed, as it happens with the present PHB matrix. Thus, the main crystallization exotherm can be seen in neat PHB with a peak crystallization temperature, T_c_, of around 115 °C, and for the composites (similarly to the behavior of T_max_^DTGA^) there is an initial increase on passing from PHB to PHBSBA2, PHBSBA4 and PHBSBA8, decreasing then for PHBSBA16. But in all cases T_c_ of the composites is higher than the one for PHB, indicating a certain nucleating effect of the mesoporous silica. At lower temperatures, and similarly to the first melting, there is a region with small exotherms, whose enthalpy increases as the SBA-15 content increases. But now it appears to be a single component in these exotherms, as observed in the amplified inset of Figure 4.

**Figure 5:**
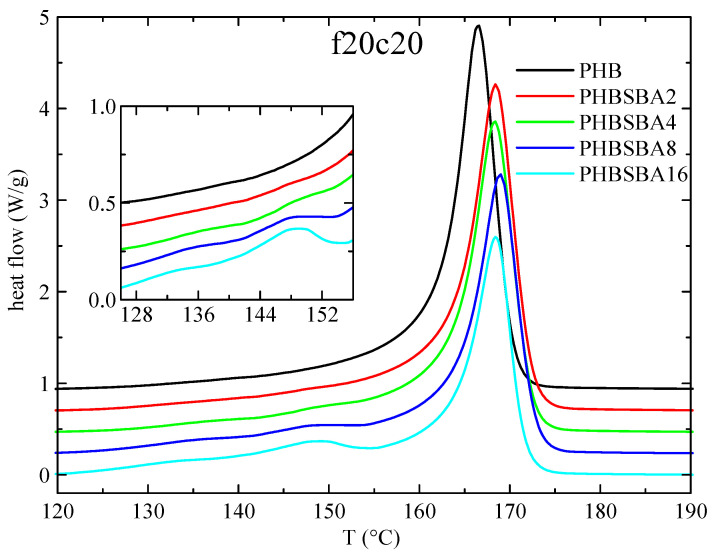
DSC heating curves (endo up) for PHB and its composites with SBA-15 in the second melting at 20 °C/min. The inset shows the amplification in the region of the melting of confined crystals of PHB.

The DSC heating curves in the subsequent second melting at 20 °C/min are presented in Figure 5. The behavior is rather similar to that in the first melting, with the eventual small melting endotherms arising from confined crystals showing also two components. Having taken into account that there is a single component in the confined crystallization, our tentative explanation for the two components of melting is that one of them arises from the confined crystals formed on cooling, while the second component is attributed to a confined recrystallization during melting.

**Figure 6:**
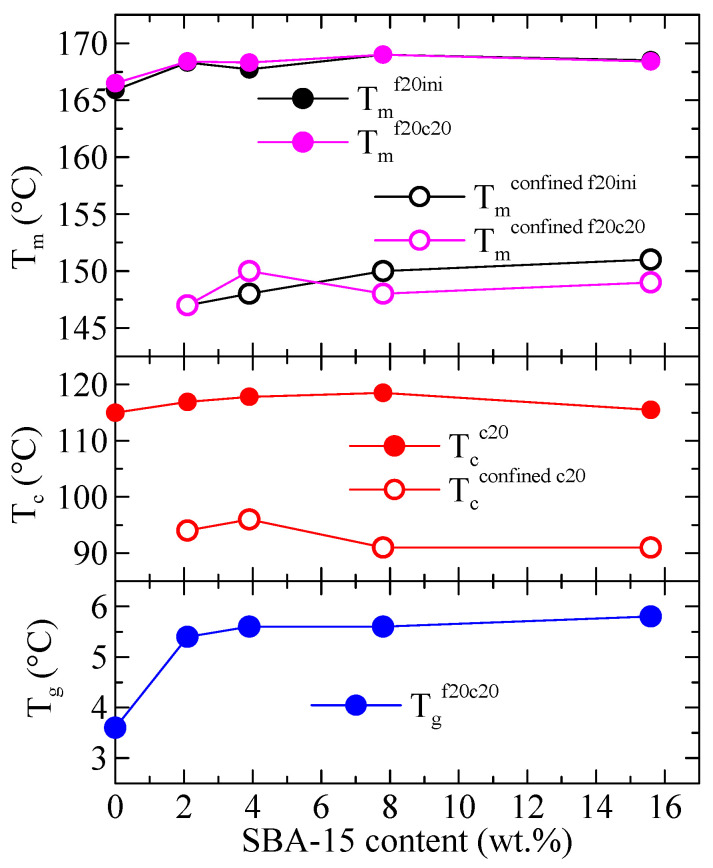
Variation with the SBA-15 content of different thermal transitions for PHB and its composites with this mesoporous silica: top: T_m_ for the first and second meltings for both the main endotherm and the confined component; middle: T_c_ for the cooling from the melt for both the main exotherm and the confined component; bottom: T_g_ for the second melting.

Variation with the SBA-15 content of the different thermal transitions observed in Figure 3, Figure 4 and Figure 5 is represented in Figure 6 for PHB and its composites. The differences among samples are almost not significant, although an increase in passing from PHB to the composites is usually observed, more evident at low SBA-15 amounts. More clear differences are deduced for the values of T_g_ (not shown in Figure 3, Figure 4 and Figure 5) where a significant increase from neat PHB to the composites is well evident.

**Figure 7:**
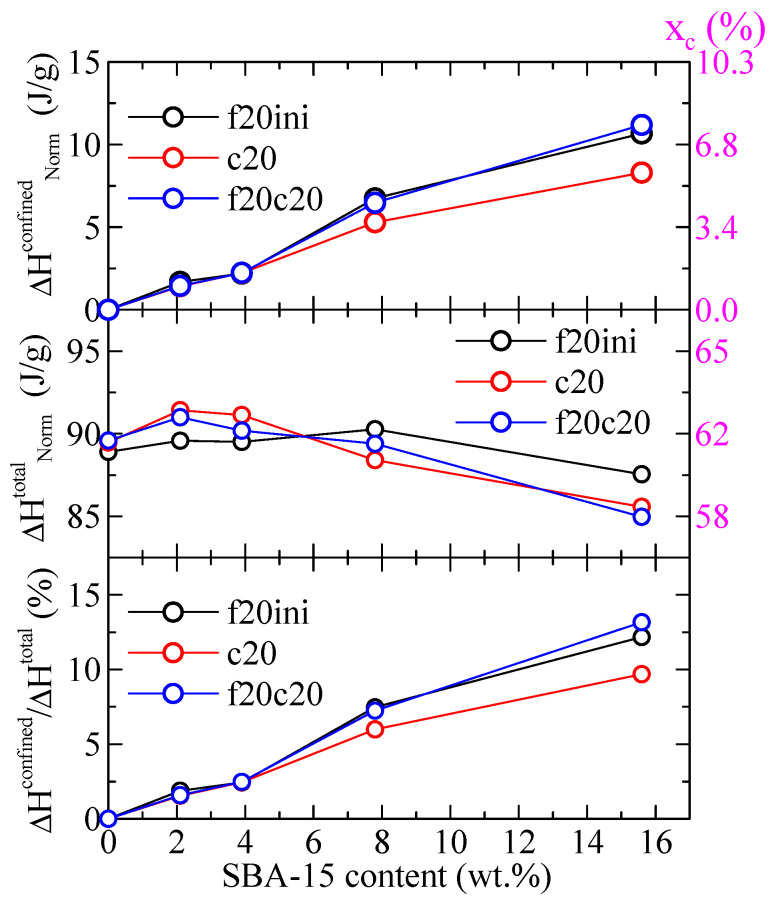
Variation with the SBA-15 content of different parameters for PHB and its composites with this mesoporous silica: top: enthalpy for the confined component for the indicated DSC ramps; middle: total enthalpy for the indicated DSC ramps; bottom: percentage of the enthalpy of the confined components over the total values. All enthalpies were normalized to the actual PHB content in the specimen. The corresponding degree of crystallinity is indicated in the right axes.

Regarding the values of the enthalpies involved, Figure 7 shows the variation with the SBA-15 content for different parameters in the PHB and its composites both the enthalpy for the confined component and the total enthalpy, for the different DSC ramps, and normalized to the actual PHB content in the specimen. It can be observed that the total enthalpy first increases with the SBA-15 content, and then reduces at the higher silica contents. The enthalpy for the confined components, however, increases markedly with the SBA-15 content (top frame in Figure 7), in such a way that the percentage of that enthalpy over the total one reaches values as considerable as 12–13%. Obviously, that increase shall be a consequence of the more total volume of silica channels available for the confinement of PHB chains as SBA-15 content increases.

The values indicated in the right axis of Figure 7 represent the corresponding percentage of crystallinity, x_c_, estimated by considering a melting enthalpy of the 100% crystalline PHB of 146 J/g [24]. As observed, a total crystallinity of around 58–62% is deduced, and the crystallinity involved in the confined crystal reaches values as high as around 6–8%.

### 3.4. X-ray Experiments with Synchrotron Radiation

**Figure 8:**
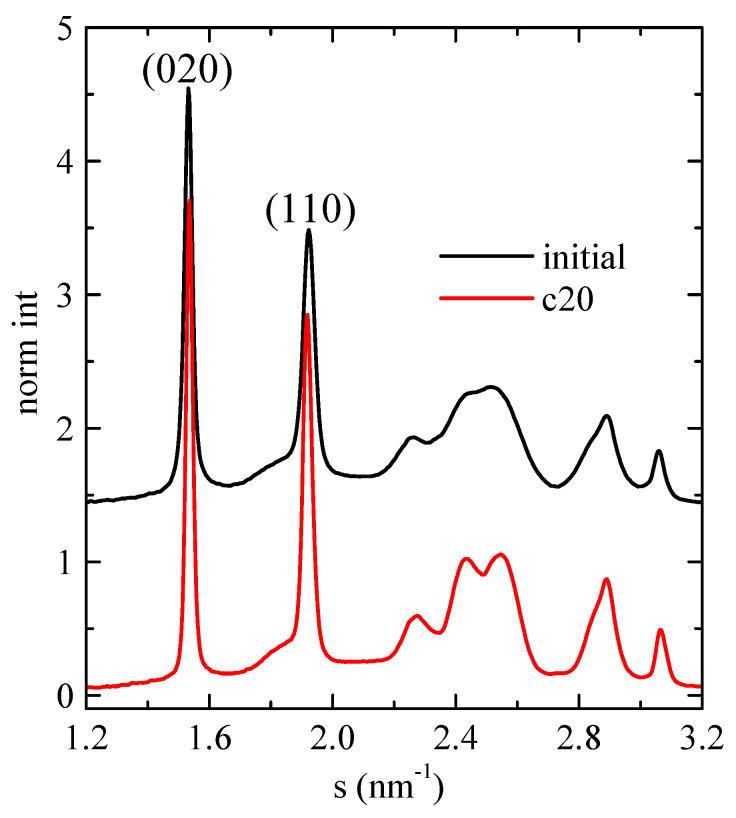
Room-temperature synchrotron WAXS diffractograms for neat PHB, showing the profile of the initial compressed-molded specimen and that after cooling from the melt at 20 °C/min.

As mentioned above, SAXS experiments turned out a very useful and conclusive tool for analyzing the confinement in composites where mesoporous SBA-15 or MCM-41 particles were involved, independently of the nature of the polymeric matrix, since the intensity of the main (100) diffraction of the mesoporous silica shows a notable discontinuity. Therefore, synchrotron X-ray experiments were performed on selected samples. Firstly, Figure 8 shows the room-temperature WAXS diffractograms for neat PHB, displaying the profile of the initial compressed-molded specimen and that after cooling from the melt at 20 °C/min. In both cases, the usual α modification of PHB is observed [6,24,33,34], with the two main diffractions corresponding to planes (020) and (110). Moreover, the diffractions corresponding to the sample crystallized from the melt at 20 °C/min are slightly narrower than those of the initial compression-molded specimen, indicative of more perfect crystals in the former.

**Figure 9:**
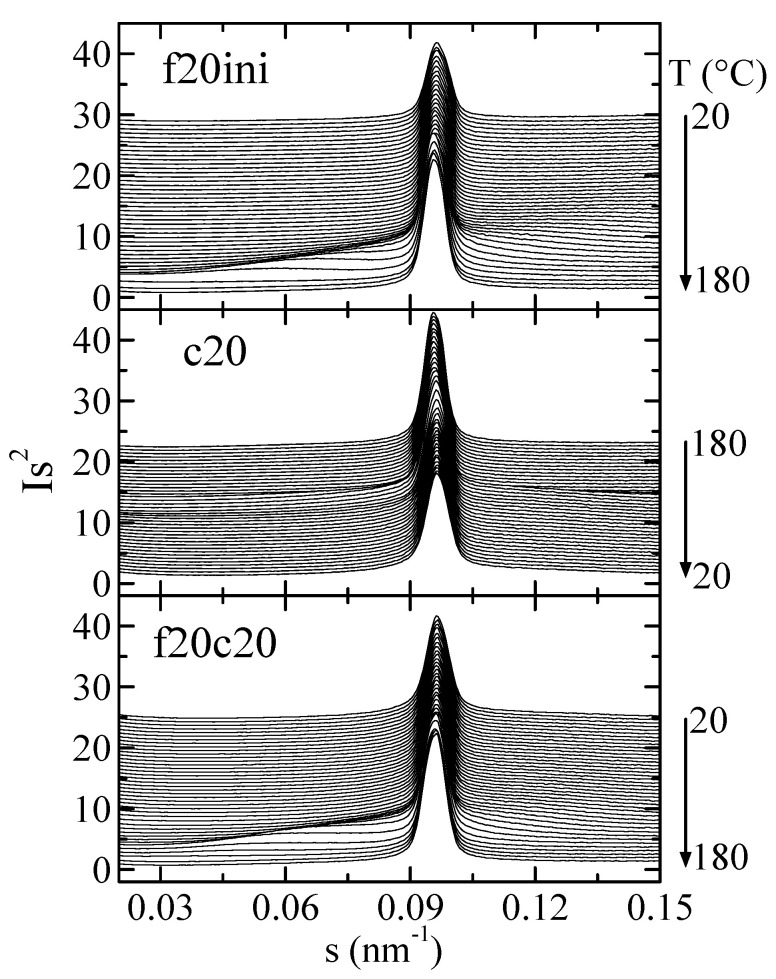
Lorentz-corrected SAXS profiles for composite PHBSBA8 during the first melting at 20 °C/min (**top**), the cooling from the melt at 20 °C/min (**middle**), and the subsequent second melting (**bottom**). Only one out of every two frames is plotted, for clarity.

Figure 9 represents the Lorentz-corrected SAXS profiles for composite PHBSBA8 during the first melting, the cooling from the melt, and the subsequent second melting. In the selected region, two features are clearly observed: first, the prominent (100) diffraction of SBA-15 [8,9,12], and second, the wide long spacing peak from the PHB crystals, which appears overlapped to the SBA-15 signal.

**Figure 10:**
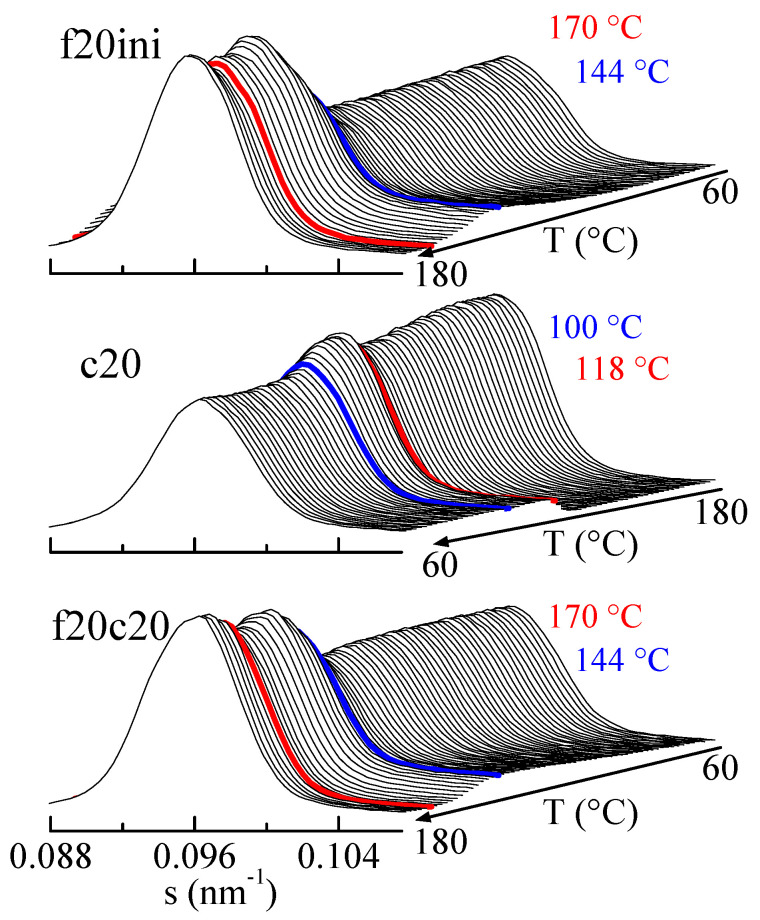
Lorentz-corrected SAXS profiles for composite PHBSBA8, showing the amplified region of the SBA-15 main reflection, during the first melting (**top plot**), the cooling from the melt (**middle plot**), and the subsequent second melting (**bottom plot**). The blue and red highlighted frames correspond to the events (crystallization or melting) arising from the confined component and the main one, respectively.

The amplification of the SBA-15 main reflection is rather informative, as observed in Figure 10. Focusing the attention on the first melting (top frame in Figure 10), it is seen that after an initial rather constant intensity of the SBA-15 diffraction, there is a pronounced increase centered at around 144 °C (blue diffractogram), and a much smaller change at around 170 °C (red diffractogram). Compared with the DSC results, these changes correspond to the melting of the confined crystals and to the main melting, respectively, but now the rather relevant issue is that confinement is much better observed than the total melting, contrary to the case in the DSC results.

Similar arguments apply for the cooling from the melt (middle representation in Figure 10) and for the second melting (lower plot in Figure 10), where the decrease in the cooling and the increase in the heating are much greater for confinement than for main crystallization or main melting.

As mentioned above, these variations in the intensity of SBA-15 diffraction depend on the scattering contrast between the walls and the inside of the SBA-15 channels before and after the melting of the confined polymer chains (and also, evidently, on the amount of pore filling).

**Figure 11:**
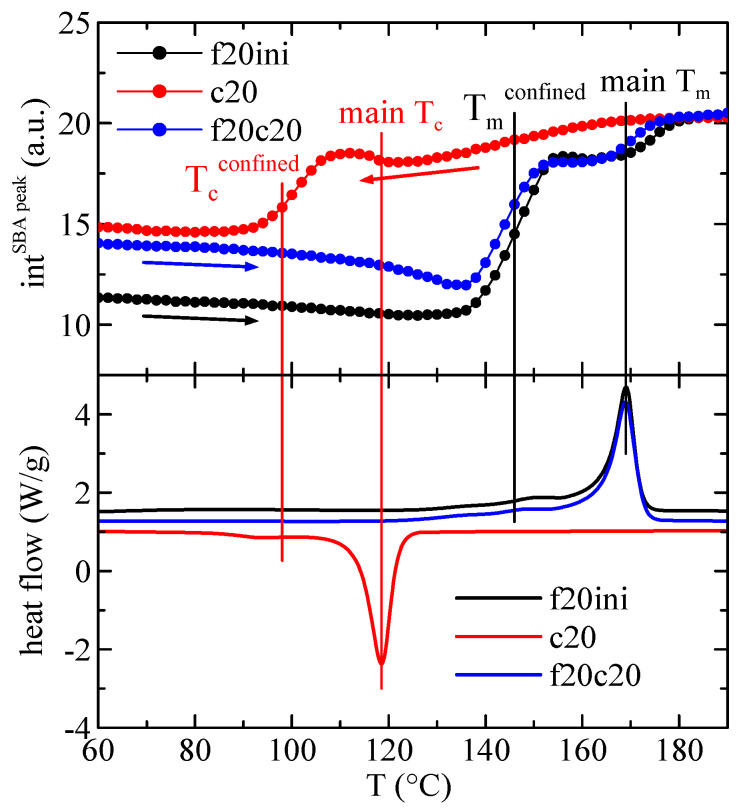
Variation with temperature of the intensity of the main SBA-15 diffraction in composite PHBSBA8 (**upper**) during the first melting (f20ini) (black arrow), cooling (c20) (red arrow) and second melting (f20c20) (blue arrow) processes, compared with the corresponding DSC curves (**lower**). The straight lines indicate the different thermal events.

The quantification of the results in Figure 10 regarding the intensity of the main SBA-15 diffraction is represented in Figure 11, showing, in the upper frame, the variation with temperature of that intensity in composite PHBSBA8 during the first melting (f20ini), cooling (c20) and second melting (f20c20) processes, and compared with the corresponding DSC curves (lower plot). The concordance of the transition temperatures for the different events, indicated by the straight lines, is excellent among the two techniques. Moreover, it is well evident that confinement is much better observed through the variation of the intensity of the SBA-15 peak.

### 3.5. Dynamic Mechanical Thermal Analysis (DMTA) Experiments

**Figure 12:**
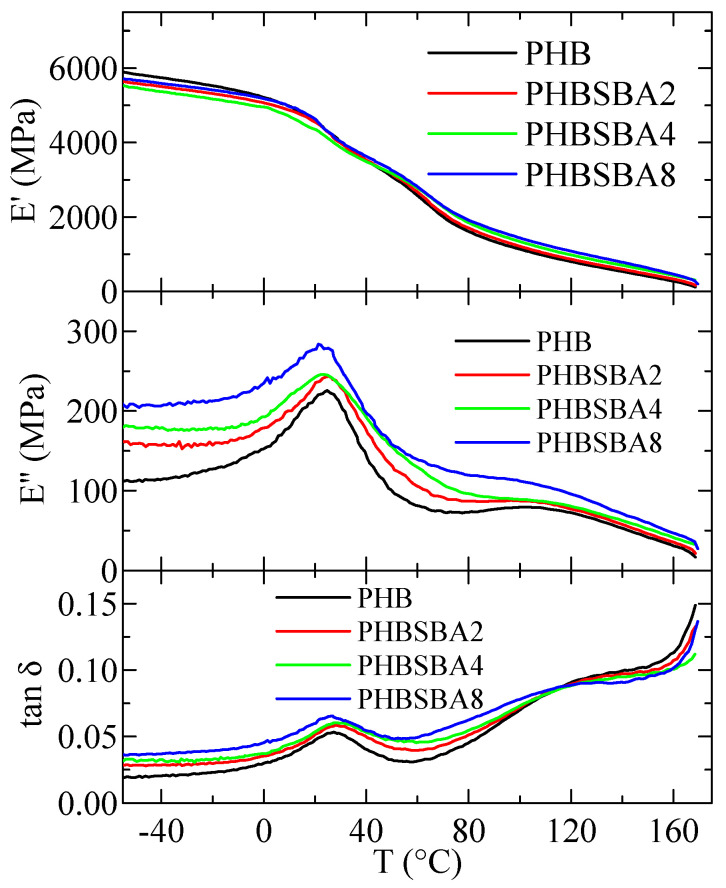
Variation with temperature of storage modulus, E′, loss modulus, E″, and tan δ curves (at 3 Hz) for PHB and its composites with SBA-15.

Figure 12 shows the variation in temperature of storage modulus, E′, loss modulus, E″, and tan δ curves (at 3 Hz) for PHB and its composites with SBA-15. Two relaxations, named α and β in order of decreasing temperatures are observed in the E″ and tan δ curves. Sample PHBSBA16 was too brittle and could not be analyzed by DMTA.

There have been different studies about the relaxation behavior in PHB. Some of them are based on DMTA measurements [1,35], and others refer to electrical analysis [36,37] or piezoelectricity [38,39]. Another relaxation was reported at temperatures lower than those analyzed here. That lowest-temperature relaxation, designated as γ, was attributed by some authors [35] to the water absorbed in PHB, but more recent works [36,37] are in favor of associating its origin to motions of the ester groups, as it happens in other polyesters.

Regarding the here named β relaxation, there is clear consensus being ascribed to the glass transition [1,35], while the one located at the highest temperature, the α relaxation, is attributed to movement within the crystalline regions as they become soft due to their partial melting.

**Figure 13:**
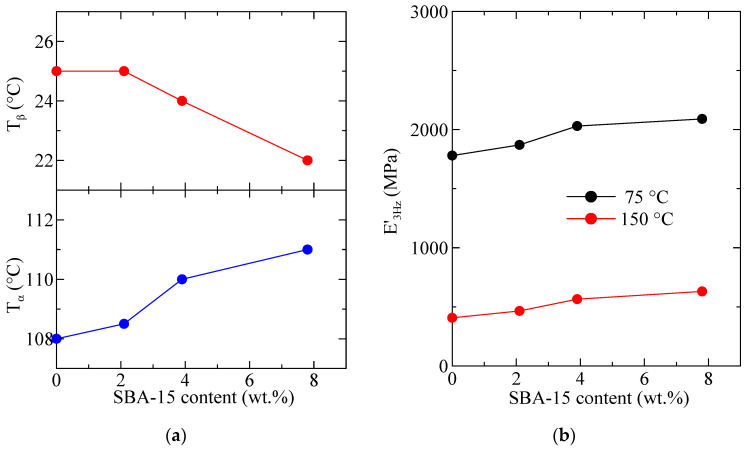
Variation with the SBA-15 content, for PHB and its composites, of (**a**) the temperature location of the α and β relaxations (E″ basis at 3 Hz); (**b**) the values of storage modulus at two different representative temperatures.

The variation with the SBA-15 content, for PHB and its composites, of the temperature location of the α and β relaxations (E″ basis at 3 Hz) is shown in Figure 13a. It can be observed that the temperature for the α relaxation increases with the silica content, while that for the β relaxation (the glass transition) decreases. This behavior is different from the one deduced from the DSC experiments, although values reported in Figure 6 (bottom frame) correspond to the ones derived from the melting after crystallization at 20 °C/min and not to the initial films. This discrepancy could be associated with differences in the residual secondary crystallization (which is a detrimental characteristic in the PHB polymer) between the pure matrix and the distinct composites. Specimens for DMTA have remained at a certain time at room temperature before these measurements were performed. Thus, the secondary crystallization of PHB chains could take place, occurring at a higher extent in the pristine polymer than in the composites, pointing out another positive role of the presence of mesoporous SBA-15 particles. Nevertheless, determination by DSC in the second heating process does not show any change in the crystalline regions, so mobility within the amorphous phase does not allow further cold crystallization. On the other hand, the β process, ascribed to the glass transition, is located in the DMTA experiments at considerably higher temperatures than that obtained from the DSC ones, as usual [40,41].

Figure 13b shows the variation in the silica content of the values of storage modulus at two different representative temperatures. Ideally, the best temperature to consider is 25 °C (ambient conditions) but, as observed in Figure 12, this temperature lies in the middle of the β relaxation (T_g_), which is not convenient. Thus, the following representative temperatures were chosen: 75 °C: in between T_β_ and T_α_, and 150 °C: above T_α_. It can be observed that at these two temperatures (both above T_g_) the rigidity increases with the SBA-15 content. The reinforcement role of silica is mainly played as the polymeric matrix is softened, and values of storage moduli increase as the SBA-15 content does.

## 4. Conclusions

Several composites based on poly(3-hydroxybutyrate) (PHB) and mesoporous SBA-15 silica were prepared by solvent-casting, followed by compression molding. The thermal properties and crystalline details of these composites were studied, paying special attention to the confinement of polymeric chains of PHB into the pores of the silica.

TGA measurements, under nitrogen, allow determining the actual SBA-15 content in the samples and also indicate slightly higher thermal stability in the composites. In fact, the value of T_max_^DTGA^ increases first in composites PHBSBA2 and PHBSBA4, then decreases for the other two composites, although the values of T_max_^DTGA^ are always higher than the ones for neat PHB.

Confinement was analyzed first by DSC and it was confirmed by SAXS experiments, which show a notable discontinuity in the intensity of the first-order (100) diffraction of the mesoporous silica. The rather relevant issue is that in the DSC results the exo- or endotherm related to confinement is much smaller than the main peaks, while in the SAXS results the variation of the intensity of the silica diffraction is considerably higher for confinement than for the total melting or crystallization.

In addition, WAXS diffractograms indicate that the usual α modification of PHB is observed in all samples, either neat PHB or its composites.

DMTA results show, in the temperature interval analyzed, two relaxations, named α and β in order of decreasing temperatures, in all specimens. Sample PHBSBA16 is too brittle and cannot be analyzed by DMTA. The temperature for the α relaxation increases with the silica content, while that for the β relaxation (the glass transition) decreases. The role of silica as a filler is mainly observed at temperatures higher than the relaxation mechanism ascribed to generalized motions within the amorphous regions, i.e., the glass transition. In such cases, stiffness is dependent on SBA-15 content.

## Figures and Tables

**Table 1:** TGA results under nitrogen for PHB and its composites, prepared by melt extrusion: SBA-15 content (determined at 650 °C) and temperature at the maximum in the DTGA curves (T_max_^DTGA^).

Specimen	SBA-15 Content (wt.%)		T_max_^DTGA^ (°C)
	Nominal	From TGA	
PHB	0	0	283.2
PHBSBA2	2	2.1	287.2
PHBSBA4	4	3.9	292.8
PHBSBA8	8	7.8	285.8
PHBSBA16	16	15.6	284.3

## Data Availability

Data are contained within the article.
